# Pharmacokinetics and Safety Studies in Rodent Models Support Development of EPICERTIN as a Novel Topical Wound-Healing Biologic for Ulcerative Colitis[Fn fn4]

**DOI:** 10.1124/jpet.121.000904

**Published:** 2022-03

**Authors:** Daniel Tusé, Micaela Reeves, Joshua Royal, Krystal T. Hamorsky, Hanna Ng, Maria Arolfo, Carol Green, Abhishek Trigunaite, Toufan Parman, Goo Lee, Nobuyuki Matoba

**Affiliations:** GROW Biomedicine, LLC and DT/Consulting Group, Sacramento, California (D.T.); Department of Pharmacology and Toxicology (M.R., J.R., N.M.), Department of Medicine (K.T.H.), and James Graham Brown Cancer Center, Center for Predictive Medicine (K.T.H., N.M.), University of Louisville, Louisville, Kentucky; SRI Biosciences Division, SRI International, Menlo Park, California (H.N., M.A., C.G., A.T., T.P.); and Department of Pathology, University of Alabama at Birmingham, Birmingham, Alabama (G.L.)

## Abstract

The novel wound-healing biologic EPICERTIN, a recombinant analog of cholera toxin B subunit, is in early development for the management of ulcerative colitis. This study established for the first time the pharmacokinetics (PK), bioavailability (BA), and acute safety of EPICERTIN in healthy and dextran sodium sulfate-induced colitic mice and healthy rats. For PK and BA assessments, single administrations of various concentrations of EPICERTIN were given intravenously or intrarectally to healthy and colitic C57BL/6 mice and to healthy Sprague-Dawley rats. After intravenous administration to healthy animals, the drug’s plasma half-life (*t*_1/2_) for males and females was 0.26 and 0.3 hours in mice and 19.4 and 14.5 hours in rats, respectively. After intrarectal administration, drug was detected at very low levels in only four samples of mouse plasma, with no correlation to colon epithelial integrity. No drug was detected in rat plasma. A single intrarectal dose of 0.1 µM (0.6 µg/mouse) EPICERTIN significantly facilitated the healing of damaged colonic epithelium as determined by disease activity index and histopathological scoring, whereas 10-fold higher or lower concentrations showed no effect. For acute toxicity evaluation, healthy rats were given a single intrarectal administration of various doses of EPICERTIN with sacrifice on Day 8, recording body weight, morbidity, mortality, clinical pathology, and gross necropsy observations. There were no drug-related effects of toxicological significance. The no observed adverse effect level (intrarectal) in rats was determined to be 5 µM (307 µg/animal, or 5.2 µg drug/cm^2^ of colorectal surface area), which is 14 times the anticipated intrarectally delivered clinical dose.

**SIGNIFICANCE STATEMENT:**

EPICERTIN is a candidate wound-healing biologic for the management of ulcerative colitis. This study determined for the first time the intravenous and intrarectal pharmacokinetics and bioavailability of the drug in healthy and colitic mice and healthy rats, and its acute safety in a dose-escalation study in rats. An initial therapeutic dose in colitic mice was also established. EPICERTIN delivered intrarectally was minimally absorbed systemically, was well tolerated, and induced epithelial wound healing topically at a low dose.

## Introduction

Topical mucosal administration of cholera toxin B subunit (CTB), such as via oral delivery or intrarectal enema, leads to broad-spectrum biologic effects, including immunostimulatory effects as well as specific immunosuppressive effects against autoimmune disorders, excess inflammation, and allergic reactions (Baldauf et al., 2015; Royal and Matoba, 2017). We previously reported that a recombinant CTB containing a C-terminal endoplasmic reticulum (ER) retention motif (CTB-KDEL), but not native CTB, induces colon epithelial wound healing in colitis via the activation of an unfolded protein response (UPR) in colon epithelial cells (Royal et al., 2019a). CTB-KDEL’s capacity to induce UPR and epithelial restitution or wound healing was corroborated in a dextran sodium sulfate-induced acute colitis mouse model (Baldauf et al., 2017; Royal et al., 2019a). Furthermore, CTB-KDEL induced a UPR and upregulated wound healing pathways and maintained viable crypts in colon explants from patients with inflammatory bowel disease (IBD) (Royal et al., 2019a). CTB-KDEL, herein named EPICERTIN (epithelial cell ER-targeted protein), exhibits unique wound healing effects in the colon that are mediated by its localization to the ER and subsequent activation of UPR in epithelial cells. These results suggest EPICERTIN’s utility as a novel therapeutic for mucosal healing, which is a significant unmet need in the management of IBD.

Here, we report for the first time the pharmacokinetics (PK), bioavailability (BA), and acute safety of EPICERTIN in healthy and dextran sulfate sodium-induced colitic mice and healthy (noncolitic) rats using single administrations of study drug at escalating dose levels. The results of these initial studies encourage further development of EPICERTIN as a novel candidate biologic for the topical management of IBD, especially for ulcerative colitis (UC).

## Materials and Methods

### Test Articles and Reagents

#### EPICERTIN

EPICERTIN is a pentameric protein with a molecular mass of 61.4 kDa (5 × 12.281 kDa per monomer). A 67-µM (4.11 mg/ml) solution of EPICERTIN in PBS, pH 7.4 (lot number: TSR-PD-17-007-1) served as the stock and was prepared for the Matoba Laboratory, University of Louisville, by Kentucky BioProcessing, Inc. (Owensboro, KY). Dilutions of the stock in PBS were made to reach the target test article concentrations of 1, 2, 5, and 10 µM. PBS solution (pH 7.4; Life Technologies Corp.) without drug served as the vehicle. The stock solutions were stored at 5 ± 3°C; diluted solutions were prepared on the same day of animal dosing and stored at 5 ± 3°C. Solutions were brought to ambient temperature prior to administration.

#### Dextran Sodium Sulfate

Dextran sodium sulfate (DSS) of molecular weight 36,000–50,000 Da (MP Biomedicals cat# 160110, lot# M8667) was used in these studies. Solutions of 3%, 3.5%, 4%, and 5% (w/v) DSS in drinking water were prepared for induction of colitis in the murine model in a dose range-finding study. The results of this study (see *Experimental Procedures*) suggested that 3% DSS was an optimal concentration to induce the desired effects (data not shown).

### Animal Models

#### Mouse

C57BL/6 (Envigo) mice were used in studies performed at SRI International, 45 of each sex, 10–11 weeks of age at first dose (*N* = 9/sex/treatment). Body weights of healthy mice at first dose ranged from 19.6 g to 27.8 g (males) and 16.5 g to 19.4 g (females). Weights of DSS-induced colitic mice at first dose ranged from 15.9 g to 19.9 g (males) and 12.2 g to 16.1 g (females). Drug administration was either by bolus intravenous injection into the tail vein, or by bolus topical colonic administration via intrarectal infusion. Female C57BL/6 (Jackson) mice (*N* = 12/treatment) were used in studies performed at the University of Louisville. Mice were 9–10 weeks of age at dosing. Drug administration was by bolus topical colonic administration via intrarectal infusion.

#### Rat

Sprague-Dawley (Charles River Laboratories) rats were used in this study, nine of each sex, 7–8 weeks of age at first dose. Body weights ranged from 218 g to 237 g (males) and 179 g to 186 g (females). Of the 18 animals assigned to the study, 12 were single jugular vein catheterized (JVC) and 6 were dual JVC by the vendor prior to shipment. JVC animals were employed to assess PK parameters with systemically administered drug. The remaining animals were exposed to test articles by bolus topical colonic administration via intrarectal infusion.

### Animal Care

#### Animal Welfare

Pharmacokinetics, bioavailability, and acute toxicity studies were conducted by SRI Biosciences, a division of SRI International (Menlo Park, CA), an accredited contract research organization (CRO), whereas the dose–response assessment in the DSS acute colitis mouse model was performed at the University of Louisville. General procedures for animal care and housing in these studies were in accordance with the current Association for Assessment and Accreditation of Laboratory Animal Care recommendations, current requirements stated in the Guide for the Care and Use of Laboratory Animals (National Research Council), and current requirements as stated by the U.S. Department of Agriculture through the Animal Welfare Act and Animal Welfare Regulations (July 2020). Housing, including number of animals per cage, light/dark cycles, temperature, and other environmental parameters, identification, randomization protocols, and quarantines were as approved for each species by the CRO’s institutional animal care and use committee. Studies conducted at the University of Louisville were approved by that institution’s institutional animal care and use committee.

#### Food and Water

Envigo Teklad Certified Global 18% rodent diet #2018C and LabDiet Laboratory Autoclavable Rodent Diet 5010* was provided ad libitum at SRI Biosciences and the University of Louisville, respectively. At the CRO, feed is analyzed periodically to ensure that contaminants known to be capable of interfering with the study and reasonably expected to be present in such feed are not present at levels that would affect the study. Documentation of feed analyses is maintained at the CRO for reference. Water (purified, reverse osmosis) was provided ad libitum. Based on previous reports, no contaminants that could interfere with and affect the results of the study were expected to be present in the water. Copies of annual analysis reports are maintained at the CRO for reference. For the mouse colitis model, DSS-induction of colitis was achieved by providing drinking bottled water with 3% DSS as described in *Experimental Procedures*.

### Analytical

Drug levels in mouse and rat plasma samples were quantified by a CTB sandwich ELISA method using the rat anti-CTB monoclonal antibody 7A12B3 for capture and a rabbit anti-CTB polyclonal antibody (Abcam, Cambridge, MA) for detection developed by University of Louisville (Reeves et al., 2021) and implemented by the CRO. Endpoint detection was determined using 3,3′,5,5′-tetramethylbenzidine, and absorbance was measured at 450 nm. The linear range for this assay is 9.7–400.1 ng/ml. A complete standard operating procedure may be found in the Supplemental Material. For mouse plasma, the method had a lower limit of quantitation (LLOQ) of 1.9 ng drug/ml; for rat plasma, the LLOQ was 0.457 ng drug/ml.

### Experimental Procedures

#### DSS-Induced Colitis Mouse Model

The DSS murine model of acute colitis selected for this study closely approximates the pathology of human UC (Perše and Cerar, 2012; Jiminez et al., 2015; Kiesler et al., 2015).

For the intrarectal dose–response analysis study performed at the University of Louisville, colitis was induced by treating mice with 3% DSS in drinking water for 7 days (Days 0 to 7) prior to intrarectal administration of test articles on Day 7 (see *Test Article Administration*). After test article administration, mice were allowed to recover with normal drinking water for 5 days prior to sacrifice on Day 12. Mice were housed in groups of 5/cage and received unlimited food. Daily body weights were obtained each morning. At time of body weight measurements, mice were examined for morbidity. Fecal samples were obtained upon sacrifice. Disease activity index (DAI) scores were determined as an average score encompassing body weight recovery at time of sacrifice, stool consistency, and presence of blood in stool. Histopathological damage scores were determined from H&E-stained cross sections of distal colon tissue and incorporate the following parameters: crypt architecture, inflammatory cell infiltrates, muscle thickening, goblet cell depletion, and crypt abscesses, as described previously (Reeves et al., 2021).

For the PK/toxicokinetic (TK) and safety studies performed at SRI International, colitis was induced by treating mice with 3% DSS in drinking water for 5 days (Days 0 to 4) followed by regular water for 3 days (Days 5 to 7) prior to administration of test article to Groups 4 and 5 on Day 7 (see *Test Article Administration*). A pretest was performed to confirm that the severity of colitis in these conditions was comparable to that of the DSS study performed at the University of Louisville. Mice were housed in groups of 3/cage and received unlimited food. Daily body weights were obtained each morning. At time of body weight measurements, the presence of blood in fecal matter and consistency of fecal material were monitored.

#### Test Article Administration

Mice were administered test articles intravenously by bolus injection via the tail vein. Rats were administered test articles intravenously via jugular vein catheter. The intravenous dosing groups in both species were used to establish kinetics for generation of bioavailability data (i.e., comparative uptake data). The intrarectal administration resulting in topical (luminal) exposure of the colonic mucosae to test articles was accomplished similarly in mice and rats. Immediately prior to dose administration, each animal was removed from its home cage and gently handled to stimulate any imminent defecation. Animals were not anesthetized for intrarectal administration.

A plastic flexible cannula (gavage needle 27 gauge in mice, 15 gauge in rats) was inserted intrarectally, with the catheter reaching approximately 3–4 cm proximal to the anus in mice and 8 cm proximal to the anus in rats, to ensure colonic delivery. Lubrication was applied, allowing for easier insertion, by dipping the gavage needle in neutral saline prior to inserting the gavage needle. Once fully inserted, test article solution (0.1 ml in mice; 1.0 ml in rats) was slowly dispensed and the animal positioned head-down for approximately 30–90 seconds to minimize loss of the test solution before being returned to its home-cage. In both species, male and female animals were administered equal dose volumes of test article solutions using identical procedures.

#### Sample Collection

In mice, blood was collected from the retro-orbital sinus under isoflurane anesthesia into tubes containing sodium citrate, processed to plasma, and stored frozen at ≤−60°C. Approximately 0.3 ml whole blood (∼150 μl of plasma) was collected per sample. In the intravenous dosing group (*N* = 9/sex), blood was collected at 5, 15, and 60 minutes, and 4, 8, and 24 hours post dose. In the intrarectal dosing groups (*N* = 9/sex/treatment), blood was collected at 15 and 60 minutes, and at 2, 4, 8, and 24 hours post dose. Groups of three mice were each assigned to two timepoints; no groups underwent blood collection at two timepoints in a row.

In rats, blood was collected from the jugular vein catheter into tubes containing sodium citrate, processed to plasma, and stored frozen at −78°C to −80°C. Blood was not collected from the catheter in which drug was administered. A maximum volume of approximately 0.3 ml of whole blood (∼150 *μ*l of plasma) per sample was obtained from each animal. Samples were collected from the intravenous dosing group (*N* = 3/sex) at 5, 20, and 30 minutes, and at 1, 2, 4, 8, 24, and 48 hours post dose, and from the intrarectal dosing groups (*N* = 3/sex/treatment) at 10, 20, and 30 minutes, and 1, 2, 4, 8, 24, and 48 hours post dose.

#### Euthanasia

Mice were sacrificed by an overdose of sodium pentobarbital administered via intraperitoneal injection. Rats were sacrificed by an overdose of sodium pentobarbital administered via intravenous injection in the PK study and by intraperitoneal injection in the acute toxicity study.

#### PK/TK Analysis

Drug levels in mouse and rat plasma were evaluated using the Phoenix WinNonlin (version 6.4) software to perform noncompartmental data analysis. The following parameters and constants were determined: maximal observed plasma concentration (C_max_), observed time to maximum plasma concentration (T_max_), area under the plasma concentration time curve to the last time point and extrapolated to infinity (AUC_last_ and AUC_inf_), and terminal elimination half-life (*t*_1/2_). C_max_ and AUC_last_ for results in mice are presented as the mean and S.E. PK/TK parameters for results in rats were calculated for each individual animal and are presented as the mean and S.D.

#### Toxicology

Rats in the toxicology arm of the study were administered PBS vehicle or EPICERTIN at several concentrations intrarectally on Day 1 (*N* = 3/sex/treatment) and sacrificed on Day 8. Clinical observations were recorded once daily approximately 2–4 hours post dose and prior to necropsy. Animals were examined for any altered clinical signs, including evaluation of the rectal area for evidence of any test article material (exclusive of normal fecal material) that may have been discharged and of any rectal irritation, gross motor and behavioral activity, and observable changes in appearance. Body weights were recorded twice per week and on Day 8 prior to necropsy. Blood was collected from the retro-orbital sinus of rats under 60% CO_2_/40% O_2_ anesthesia for clinical pathology evaluation on Day 3. Hematology samples were collected using K_3_EDTA as the anticoagulant. No anticoagulant was used for clinical chemistry samples.

Hematology measurements included hematocrit, hemoglobin, red blood cell count, red blood cell distribution width, white blood cell count, white blood cell differential and absolute counts, mean corpuscular hemoglobin, mean corpuscular volume, mean corpuscular hemoglobin concentration, platelet count, mean platelet volume, absolute reticulocyte, and percent reticulocyte.

Clinical chemistry measurements included total bilirubin, creatinine, sodium, potassium, chloride, cholesterol, triglyceride, glucose, blood urea nitrogen, aspartate aminotransferase, alanine aminotransferase, alkaline phosphatase, calcium, phosphorus, total protein, albumin, albumin/globulin ratio, and globulin.

Macroscopic evaluation included external examination of all body orifices and an examination of all cranial, thoracic, and abdominal organs (including colon).

### Statistical Analyses

Body weight and clinical pathology data were evaluated by one-way ANOVA or Welch’s ANOVA, followed by Dunnett’s test (if the ANOVA was significant). All other numeric parameters were evaluated by Student’s *t* test, unless specified otherwise. If appropriate, other post hoc analyses were also performed. For clinical pathology data, values for parameters that were not within the detection threshold were not included in the statistical evaluation. The criterion for null hypothesis rejection was *P* ≤ 0.05.

## Results

### Dose–Response Assessment of EPICERTIN in a DSS Colitis Mouse Model

**Fig. 1.**
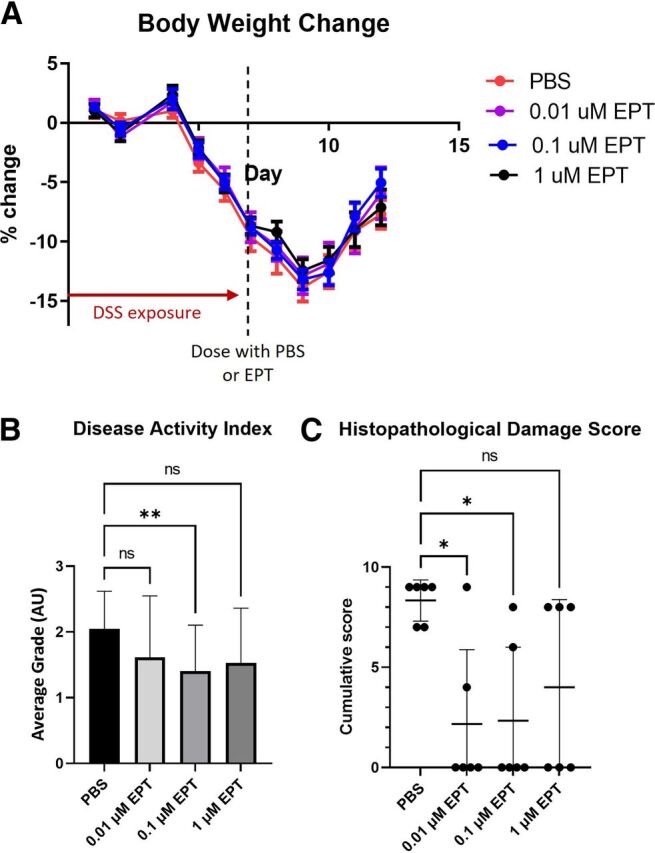
Dose–response analysis of intrarectally administered EPICERTIN. Mice were topically (colonically) treated with 0.01 μM, 0.1 μM, or 1 μM EPICERTIN (EPT) or PBS vehicle control (*N* = 12/treatment) at the end of DSS exposure (Day 7) and euthanized for analysis on Day 12. (A) Body weight change from baseline of each treatment group in the DSS acute colitis study. There was no significant difference between groups (two-way ANOVA). (B) DAI scores of each treatment group in the DSS acute colitis study. DAI is an average score consisting of body weight, stool consistency, and blood in stool. (C) Histopathological damage scores of each treatment group in the DSS acute colitis study. Histopathological damage score is a cumulative score consisting of crypt architecture, inflammatory infiltrates, muscle thickening, and goblet cell depletion. Mean ± S.D. is displayed. *P < 0.05, **P < 0.01; Welch’s ANOVA with Dunnett’s multiple comparisons post-test.

To evaluate the efficacy of EPICERTIN and determine an optimal target dose for a first-in-human clinical study, a dose–response analysis was performed using an acute DSS colitis mouse model. Female C57BL/6 mice (8 weeks of age) were exposed to 3% DSS ad libitum in drinking water for 7 days. Immediately upon DSS cessation, mice were administered intrarectally 100 μl of either PBS (vehicle and procedural control) or 0.01, 0.1, or 1 μM EPICERTIN (corresponding to 0.0614 μg, 0.614 μg, or 6.14 μg of drug per animal, respectively). All animals were monitored for an additional 5 days following dose administration to determine disease activity index (DAI), followed by postmortem histologic examination of colonic tissues to correlate the impact of drug dosage on reversal of colitis. None of the treatment groups had a statistically significant difference in body weight recovery compared with the PBS group; however, on Days 11 and 12, the 0.1 and 0.01 μM groups began to trend toward an increase in body weight recovery (Fig. 1A). EPICERTIN at 0.1 μM concentration (0.614 μg per animal) was the only dose that decreased DAI scores in a statistically significant manner (Fig. 1B). Although not statistically significant, treatment with 1 μM or 0.01 μM EPICERTIN slightly decreased DAI scores. Histopathological assessment was performed to corroborate the aforementioned DAI results. Similar to the DAI results, histopathological damage scores indicated that EPICERTIN treatment mitigated DSS-induced acute colitis, with 0.01 µM and 0.1 μM EPICERTIN being statistically significant compared with the vehicle control (Fig. 1C). These two dose levels led to less ulceration and decreased inflammation compared with the vehicle control. Although not statistically significant, treatment with 1 μM EPICERTIN trended to a lower histologic damage score than the PBS group.

**Fig. 2.**
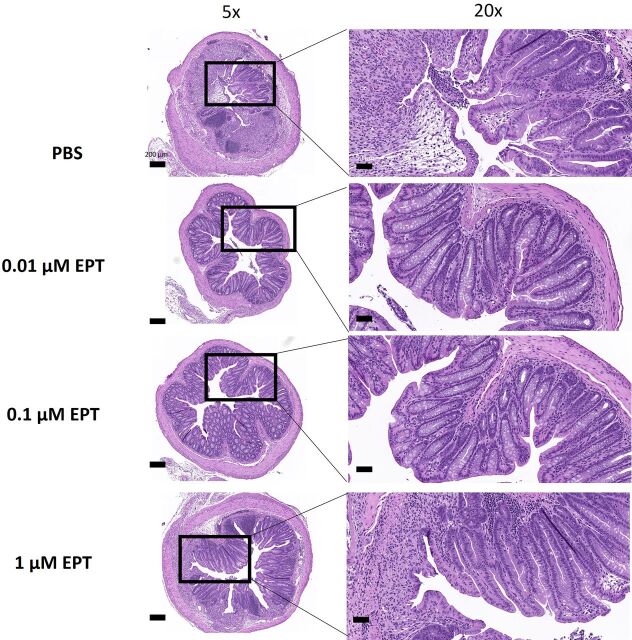
Effects of intrarectally administered EPICERTIN on murine colon histologic alterations induced by DSS. Mice in the 0.1 μM EPT dose group (0.614 μg EPT in 100 µl volume per animal) were more protected from crypt loss, inflammatory cell infiltration, and goblet cell depletion than any other group. Representative 5× (left) and 20× (right) photomicrographs of H&E-stained distal colon tissues from each treatment group. Bar = 200 µm (5×) or 50 µm (20×). *Images for PBS, 0.01 μM, 0.1 μM, and 1 μM groups have histopathological damage scores of 9, 0, 6, and 8, respectively.

A further in-depth histologic examination of the distal colon revealed that treatment with 0.1 μM EPICERTIN decreased the prevalence and incidence of crypt loss and structural alterations, immune cell infiltrates, and goblet cell depletion compared with 0.01 μM or 1 μM drug-treated and PBS groups (Fig. 2). Dosing with 0.01 *μ*M EPICERTIN protected from goblet cell depletion and immune cell infiltrates; however, crypt loss and structural alterations were more common in these tissues than in the 0.1 μM EPICERTIN group (Fig. 2). Treatment with 1 μM EPICERTIN decreased incidences of crypt loss, immune cell infiltrates, and goblet cell depletion compared with control, but these effects were much less pronounced than in the 0.01 μM and 0.1 μM EPICERTIN dosing groups (Fig. 2).

### Pharmacokinetics of EPICERTIN in Healthy and Colitic C57BL/6 Mice

The design of the study arm to evaluate PK parameters when test articles were administered to male and female mice via the intravenous and intrarectal routes is shown in Table 1. Healthy (noncolitic) and colitic animals were included in the intrarectal groups to assess whether topically applied drug might be absorbed differentially into systemic circulation as a function of the integrity of the colonic epithelium.

**TABLE 1:** Experimental design of the PK study in healthy and colitic C57BL/6 mice

Group	Dosing Route	Dose (µg/mouse)	Dose Conc.(µM)	Dose Volume(ml/mouse)	No. of Normal Animals*^a^*	No. of DSS Colitis Animals*^a^*^,^*^b^*	Blood Collection Timepoints*^c^*
1	i.v.	6.14	1	0.1	9M/9F		5, 15, and 60 min, and at 4, 8, and 24 h
2	i.r.	6.14	1	0.1	9M/9F		15 and 60 min, and at 2, 4, 8, and 24 h
3	i.r.	61.4	10	0.1	9M/9F		15 and 60 min, and at 2, 4, 8, and 24 h
4	i.r.	6.14	1	0.1		9M/9F	15 and 60 min, and at 2, 4, 8, and 24 h
5	i.r.	61.4	10	0.1		9M/9F	15 and 60 min, and at 2, 4, 8, and 24 h

*^a^*Blood was collected from 3 animals/sex for each time point and from 3 animals/sex untreated mice for baseline control samples from healthy and colitic mice.

*^b^*DSS colitis was induced by treating mice with 3% DSS in drinking water for 5 days (Days −8 to −4) followed by regular water for 3 days (Days −3 to −1) prior to the dose administration on Day 1.

*^c^*Two blood samples were collected from each mouse; three mice were assigned for each timepoint.

#### Clinical Observations

Clinical findings were observed in DSS-induced colitic mice of groups 4 and 5 (Table 1) (dosing at 6.14 μg and 61.4 μg/mouse, respectively) on Days 1 and 2. Mice of group 4 showed slight to extreme dehydration (6 male/3 female), slight to extreme hunched posture (9 male/9 female), slight to extreme hypoactivity (6 male/5 female), slight to moderate hypothermia (1 male/1 female), slight to moderate ruffled fur (6 male/3 female), skin and fur discoloration (1 female), slight to moderate squinting from both eyes that appeared to be from blood sample collection (1 male/1 female), and slight thin appearance (1 male). Similar findings including slight to moderate dehydration (4 male/4 female), moderate diarrhea (1 male), slight to moderate hunched posture (9 male/9 female), slight to moderate hypoactivity (4 male/6 female), slight to moderate hypothermia (3 male/1 female), slight ruffled fur (9 male/6 female), and slight thin appearance (1 male/6 female) were observed in group 5 (Table 2). These findings are symptoms associated with DSS treatment (Clapper et al., 2007; Perše and Cerar, 2012; Jiminez et al., 2015), were anticipated, and were not considered test article related.

**TABLE 2:** Clinical observations summary of the PK study in healthy and colitic C57BL/6 mice

Observation Type From Day 1 (Start Date) to 2 (Start Date)	Group 4 1 µM ir Colitis		Group 5 10 µM ir Colitis	
	Male	Female	Male	Female
**Dehydration**				
Number of animals affected	6	3	4	4
First to last seen	1–2	1–2	1–2	1–2
**Diarrhea**				
Number of animals affected	0	0	1	0
First to last seen			1–2	
**Eschar Formation**				
Number of animals affected	0	0	0	0
First to last seen				
**Hunched Posture**				
Number of animals affected	9	9	9	0
First to last seen	1–2	1–2	1–2	1–2
**Hypoactivity**				
Number of animals affected	6	5	4	6
First to last seen	1–2	1–2	1–2	1–2
**Hypothermia**				
Number of animals affected	1	1	3	1
First to last seen	2–2	2–2	2–2	2–2
**Ruffled Fur**				
Number of animals affected	6	3	9	6
First to last seen	1–2	1–2	1–2	1–1
**Skin and Fur Discolored**				
Number of animals affected	0	1	0	0
First to last seen		2–2		
**Squinting**				
Number of animals affected	1	1	0	0
First to last seen	1–1	1–1		
**Thin**				
Number of animals affected	1	0	1	6
First to last seen	1–1		1–1	1–2

#### Body Weights

Body weights were measured on Day 1 prior to dose administration. Body weights from DSS-induced colitic mice of groups 4 and 5 (Table 1) were significantly lower (by 20–23%) compared with healthy controls on Day 1 prior to dose administration. This reduced body weight was consequential to DSS treatment (Perše and Cerar, 2012) and was expected in the colitic animals.

#### Plasma Drug Levels

**Fig. 3.**
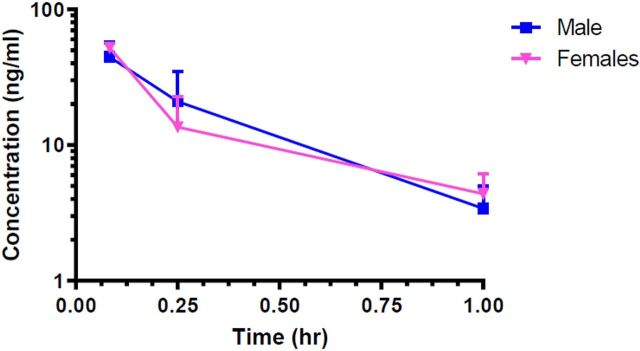
Plasma levels of EPICERTIN (intravenous administration of 6.14 μg/mouse) in healthy (noncolitic) C57BL/6 mice. Plasma concentrations of EPICERTIN were measured after a single intravenous dose of 6.14 μg/mouse (*N* = 18; 9 male/9 female) in blood samples collected at 0.0833, 0.25, and 1 hour. At 4 hours, the plasma concentrations were less than the assay LLOQ (1.9 ng/ml) in mice treated by intravenous administration. The exposure to EPICERTIN based on AUC values were similar in male and female mice; AUC_inf_ was 20.4 hours·ng/ml (males) and 20.5 hours·ng/ml (females).

The plasma concentrations of EPICERTIN in group 1 (Table 1), intravenous administration of 6.14 μg/mouse (0.263 μg/kg and 0.340 μg/kg, for males and females, respectively) are presented in Fig. 3. Drug concentrations were below the lower limit of quantitation (1.9 ng/ml) by 4 hours. Group 2–5 animals were administered EPICERTIN by the intrarectal route. Of the 72 mice administered EPICERTIN via the intrarectal route, quantifiable drug concentrations were found in only four plasma samples collected from these animals, namely, group 2, male 019 at 0.25 hour (2.22 ng/ml); group 2, male 022 at 1 hour (9.09 ng/ml); group 3, female 048 at 0.25 hour (110.9 ng/ml); and group 5, female 089 at 0.25 hour (2.17 ng/ml). All samples collected from untreated mice were below the assay’s LLOQ.

The results of the pharmacokinetic data analysis of plasma concentrations of EPICERTIN after a single intravenous dose are presented in Table 3. The observed T_max_ was 0.0833 hours (i.e., 5 minutes), measured at the first blood collection point, and the plasma concentrations at that time point were 44.6 ± 7.16 ng/ml (males) and 52.2 ± 0.944 ng/ml (females). Exposure to EPICERTIN was similar for both males and females; AUC_last_ was 19.2 ± 4.60 hours·ng/ml (males) and 18.6 ± 2.43 hours·ng/ml (females). The AUC_inf_ was 20.4 hours·ng/ml and 20.5 hours·ng/ml, for males and females, respectively. Insufficient data were available from animals administered drug intrarectally to calculate PK parameters because most plasma drug levels in the samples were below the assay’s LLOQ.

**TABLE 3:** PK of EPICERTIN in C57BL/6 mice after a single intravenous administration

Sex	Dose (μg/kg)	Route	*t*_1/2_(h)	*T*_max_(h)	*C*_max_ (ng/ml)		AUC_last_ (h·ng/ml)		AUC_inf_*^a^*(h·ng/ml)
					Mean	S.E.	Mean	S.E.	
Male	0.263	i.v.	0.26	0.0833	44.6	7.16	19.2	4.60	20.4
Female	0.340	i.v.	0.30	0.0833	52.2	0.944	18.6	2.43	20.5

*^a^*The degree of variability was not calculated for AUC_inf_ due to the composite-group study design in this experiment.

### Pharmacokinetics of EPICERTIN in Healthy Sprague-Dawley Rats

The design of the study arm to evaluate PK parameters when test articles were administered to male and female rats via the intravenous and intrarectal routes is shown in Table 4.

**TABLE 4:** Experimental design of the PK study in healthy (noncolitic) Sprague-Dawley rats

Group	Dosing Route	Dose (µg/rat)	Dose Conc. (µM)	Dose Volume*^a^* (ml/rat)	No. of Animals*^b^*	Blood Collection Times (h)
1	i.v.	307	5	1	3M/3F	0.083, 0.33, 0.5, 1, 2, 4, 8, 24, 48
2	i.r.	61.4	1	1	3M/3F	0.167, 0.33, 0.5, 1, 2, 4, 8, 24, 48
3	i.r.	307	5	1	3M/3F	0.167, 0.33, 0.5, 1, 2, 4, 8, 24, 48

*^a^*The dose was delivered in a constant volume of 1 ml for intravenous and intrarectal administrations and was not based on animal body weight.

*^b^*Animals were JVC for blood collection. Rats in group 1 were dual catheterized for both dosing and blood collection from different catheters.

#### Clinical and Behavioral Observations and Body Weights

All animals survived until their scheduled sacrifice. All animals behaved and appeared normal throughout the study. Animals were randomized, and body weights were measured on Day 1 prior to dose administration. There were no disparities in weight among the groups at the time of test article administration (data not shown for brevity).

#### Plasma Drug Levels

**Fig. 4.**
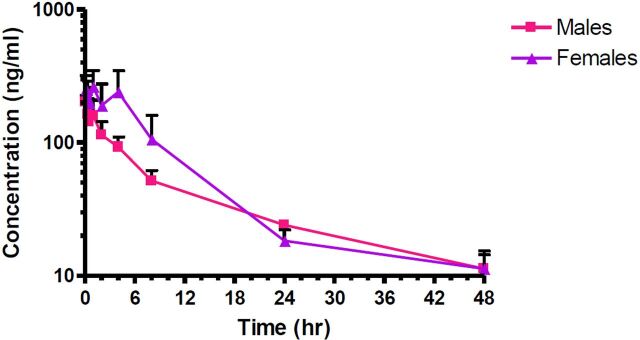
Plasma levels of EPICERTIN in healthy (noncolitic) Sprague-Dawley rats. After a single intravenous dose of 307 µg EPICERTIN (*N* = 6, 3 male/3 female), the observed mean *T*_max_ in males and females was 0.083 hours and 0.69 ± 0.53 hours, respectively. After intravenous administration, the first blood collection time has the highest observed plasma levels of the test article. The mean *C*_max_ values were 203 ± 23 ng/ml (males) and 279 ± 66 ng/ml (females). Exposure based on AUC_last_ was 1810 ± 235 hours·ng/ml (males) and 2930 ± 957 hours·ng/ml (females).

The plasma concentrations of EPICERTIN after intravenous administration of 307 μg/animal (mean of 1390 μg/kg for males and 1690 μg/kg for females) are shown in Fig. 4. The mean plasma concentrations tended to be higher in females than in males at timepoints before 24 hours and then were similar in males and females at 24 and 48 hours. The results of the PK analysis are presented in Table 5. After a single intravenous dose of 307 μg/animal, the observed mean *T*_max_ in males and females was 0.083 ± 0 hours and 0.69 ± 0.53 hours, respectively. After intravenous administration, the first blood collection time had the highest observed plasma levels of the test article. In this study, two of the female rats had *T*_max_ at 1 hour, which is atypical and may be due to poor solubility of the test article in blood, resulting in precipitation after dosing, which then steadily solubilizes, resulting in a delayed *T*_max_. The mean *C*_max_ values were 203 ± 23 ng/ml (males) and 279 ± 66 ng/ml (females). Exposure based on AUC_last_ was 1810 ± 235 hours·ng/ml (males) and 2930 ± 957 hours·ng/ml (females). AUC_inf_ values were higher but showed the same relative values for males and females. The dose of EPICERTIN was about 1.2-fold higher in females than males, but the increase in exposure based on AUC_last_ and AUC_inf_ was 1.5- to 1.6-fold higher in females. The *t*_1/2_ was long with the mean value for male rats 19.4 ± 5.20 hours and for female rats 14.5 ± 5.37 hours. PK parameters could not be calculated for groups 2 and 3 (Table 4) drug at 1 µM and 5 µM, respectively), which received the dose by intrarectal administration. Drug levels in all plasma samples collected from these groups were below the assay’s LLOQ.

**TABLE 5:** PK of EPICERTIN in Sprague-Dawley rats after a single intravenous administration (dose = 307 µg/rat)

Route	Dose*^a^* (µg/kg)	Animal	Sex	*t*_1/2_ (hr)	*T*_max_*^b^* (hr)	*C*_max_ (ng/ml)	AUC_last_ (h•ng/ml)	AUC_inf_ (h•ng/ml)	Vss^c^ (ml/kg)	CL^d^ (ml/h/kg)
										
i.v.	1400	1	M	23.2	0.083	177	1700	2170	18200	644
										
i.v.	1390	2	M	21.5	0.083	211	1650	2020	17800	687
										
i.v.	1380	3	M	13.5	0.083	222	2080	2240	9530	617
										
Mean	1390			19.4	0.083	203	1810	2140	15200	650
										
S.D.	10			5.20	0.00	23	235	112	4890	34.9
										
i.v.	1650	4	F	20.5	0.083	228	2090	2560	15500	19000
										
i.v.	1720	5	F	10.1	1	354	3970	4100	3790	6090
										
i.v.	1710	6	F	13.0	1	255	2720	2890	7240	11100
										
Mean	1690			14.5	0.69	279	2930	3180	8840	12100
										
S.D.	38			5.37	0.53	66	957	811	6020	6510
										

*^a^*Dose = 307 µg EPICERTIN delivered in 1 ml i.v. infusion per animal.

*^b^T*_max_ was generally observed at the time of first blood collection (5 min), with the exception of two female animals for which *T*_max_ was observed at 1 hour.

*^c^*Vss, steady state volume of distribution.

*^d^*CL, clearance.

### Acute Toxicity Dose Range-Finding Study in Sprague-Dawley Rats

A single-administration, dose range-finding study of EPICERTIN delivered intrarectally to male and female Sprague-Dawley rats was conducted. The objectives of this study were to determine potential toxicity of the drug when delivered topically to the colon of adult noncolitic animals, and to help establish a suitable dose level to inform subsequent nonclinical and clinical studies. The experimental design of this dose range-finding study is shown in Table 6. A PBS solution was used as the vehicle control, and three different concentrations of EPICERTIN in PBS solution were used with the goal of establishing a maximum tolerated dose and a no observed adverse effect level for the intrarectally administered drug solution.

**TABLE 6:** Experimental design of single intrarectal administration dose range-finding study in male and female Sprague-Dawley rats

Group	Treatment	Dose Conc. (µM)	Dose Volume*^a^* (ml/rat)	Dose (μg/rat)	No. of Animals (Day of Sacrifice)	
					Males (Day 8)	Females (Day 8)
1	Vehicle control	0	1	0	3	3
2	EPICERTIN	1	1	61.4	3	3
3	EPICERTIN	2	1	122.8	3	3
4	EPICERTIN	5	1	307	3	3

*^a^*The dose was delivered in a constant volume for intrarectal administrations and was not based on animal body weight.

#### Mortality/Morbidity and Clinical Observations and Body Weights

All animals survived until their scheduled sacrifice on Day 8. All animals behaved and appeared normal throughout the study. All animals exhibited normal body weight gains during the course of the study (data not shown for brevity).

#### Clinical Pathology Evaluations

*Hematology.* Blood samples were collected for analysis on Day 3. Relative to vehicle control animals (Table 6, group 1), slight, but statistically significant, increases in red blood cell distribution width were observed in male animals of group 3 (by 8%). Slight, but statistically significant, increases in absolute reticulocytes were observed in males of groups 3 and 4 (by 18% and 22%, respectively). In females, a slight decrease in percent monocytes was observed in all treated groups (by 33–48%), and this decrease was statistically significant in group 3 (by 48%) compared with animals in the control group. Absolute monocytes were also statistically significantly decreased on all treated groups (by 63%, 66%, and 56% in groups 2, 3, and 4, respectively). Group hematology results are included in Supplemental Table 1. These changes were within the normal historical range for adult Sprague-Dawley rats (Petterino and Argentino-Storino, 2006; Lee et al., 2012; Delwatta et al., 2018), were not consistent in both males and females, and therefore were considered to be of minimal toxicological significance.

#### Clinical Chemistry

Blood was collected for analysis on Day 3. A slight, but statistically significant, increase of 16% in total protein was observed in male animals of group 4 (Table 6). A slight dose-dependent increase in triglycerides (TGL) was observed in males of all treated groups (by 8–10%). In females, a slight but statistically significant decrease in glucose was observed in groups 2 and 4 (by 13% and 18%, respectively). A slight, but statistically significant, increase in albumin/globulin ratio was observed in females of group 4 (by 50%). Group clinical chemistry results are included in Supplemental Table 2. These changes were slight and within the normal historical range for adult Sprague-Dawley rats (Petterino and Argentino-Storino, 2006; Lee et al., 2012; Delwatta et al., 2018), and were considered to be of minimal toxicological significance.

#### Necropsy Observations

Group necropsy observations of major organs are summarized in Supplemental Table 3. One discolored and red mandibular lymph node was observed in one male animal in vehicle control group 1 (Table 6). Discolored and red lungs were found in one female of group 2. They were assessed by the veterinary pathologist to be a secondary effect from euthanasia. Discolored and red intestine, colon, and mucosa were observed in one female of group 3. Discolored and red intestine and jejunum were observed in one male of group 2. The necropsy findings were sporadic in control and treated groups, not correlated to drug dose, and were therefore considered to be of minimal toxicological significance.

## Discussion

The novel protein EPICERTIN, an analog of cholera toxin B subunit with unique wound-healing properties, is in early development as a candidate for the management of UC and possibly other manifestations of IBD. This study established for the first time the PK, BA, and acute safety of EPICERTIN in healthy and DSS-induced colitic mice and healthy (noncolitic) rats using single administrations of study drug at escalating dose levels.

We have previously reported results of extensive studies on EPICERTIN’s mechanism of action and its dramatic effects upon topical exposures of animal colonic tissue and surgical explants from IBD patients in vitro, and of effects in animal studies involving oral administrations of various dose levels of the drug in vivo. Specifically, we showed conclusively EPICERTIN’s wound healing and tissue restitution effects in vitro and in vivo using animal models of UC (Baldauf et al., 2017; Royal et al., 2019a; Royal et al., 2019b). Results of the present study corroborate EPICERTIN’s tissue-healing effects.

The drug concentrations used in intrarectal administrations in mice were 0.01 μM, 0.1 μM, or 1 μM EPICERTIN. In the present study, effective concentrations of EPICERTIN capable of ameliorating DSS-induced murine acute colitis ranged from 0.01 µM to 0.1 µM based on histopathological findings, with 0.1 µM appearing to be an optimal concentration on the basis of DAI (Figs. 1 and 2). Although no significant change in body weight recovery was observed, we believe that DAI scores are more representative of disease state at sacrifice as this score encompasses body weight change from baseline as well as stool consistency at time of sacrifice for each mouse. The reason for the relative lack of colitis-ameliorating response when EPICERTIN was administered intrarectally at a 10-fold higher concentration (1 µM) than the optimal dose is unclear. We speculate that it might be due to the threshold of UPR levels induced by EPICERTIN beyond which therapeutic effects start to diminish (Royal et al., 2019a). Additional insights might be gained by administering more closely spaced doses than the 10-fold drug concentrations used in this study. The mechanism(s) underlying an optimal dose–response requires further elucidation and will be the topic of subsequent studies.

Based on the PK results, EPICERTIN does not appear to be systemically absorbed into circulation after intrarectal administration. This finding applies even when the colonic epithelia are compromised in severe DSS-induced colitis. In experiments with male and female C57BL/6 mice using both normal (noncolitic) and colitic (DSS-induced) animals administered EPICERTIN intrarectally to the colon, drug levels in the majority of plasma samples (68/72 samples, or 94.4% negative) were below the assay’s LLOQ of 1.9 ng/ml in murine plasma. The lack of drug absorption was consistent regardless of the integrity of the colon, as plasma samples from intrarectally dosed noncolitic and colitic animals yielded no quantifiable drug (Table 1; group 5 colitis group). Similarly, single-administration studies in male and female Sprague-Dawley rats given EPICERTIN intravenously and intrarectally resulted in readily measurable drug plasma levels intravenously, but in no quantifiable drug in circulation from intrarectally treated animals, as all samples were below the assay’s LLOQ of 0.457 ng/ml in rat plasma. However, it is possible that the lack of measurable drug in circulation may be due to limitations of the assay. The ELISA used to quantify drug in plasma of each species has excellent sensitivity, but a low level of drug could have escaped detection. Nevertheless, results of this study showed the drug to be retained in the intestinal lumen regardless of epithelial integrity, and to exert the desired therapeutic effects. This observation, if confirmed in future work, provides a preliminary albeit important indication that topical delivery of EPICERTIN to patients suffering from UC would not necessarily lead to adverse off-target effects due to systemic absorption of the drug, even though these patients would be expected to have compromised intestinal epithelial barrier function (Schmitz et al., 1999; Pastorelli et al., 2013; Ramos and Papadakis, 2019).

Importantly, none of the doses of EPICERTIN applied in the present study led to toxicity, and the drug was well tolerated. In studies with intravenously delivered drug in both species, all animals survived until their scheduled sacrifice. All animals were normal throughout the study. Only the mice with DSS-induced colitis showed gross behavioral changes, weight changes, ruffled fur, and dehydration; these findings are symptomatic of DSS treatment and were not considered test article related. Inclusion of the colitis group was essential to understanding whether EPICERTIN is differentially absorbed into systemic circulation compared with potential drug uptake in animals with normal colons. All animals in this study survived until their scheduled sacrifice. No drug- or drug dose-related effects were observed for clinical parameters, body weights, clinical pathology, or gross necropsy observations. In this study, the MTD was not reached at the drug doses used, and the no observed adverse effect level was determined to be 5 µM (307 µg/animal; 5.2 µg drug/cm^2^ colorectal surface area). Multispecies Good Laboratory Practice (21CFR58)-compliant repeat-dose toxicity studies with recovery period are planned to support first-in-human clinical research, and those studies should provide additional perspective on safety.

The first contemplated clinical administration of EPICERTIN will be via enema. The rectal solution described herein is being co-developed with an oral formulation, which has also been shown effective in murine colitis models (Hamorsky et al., 2013; Baldauf et al., 2017; Royal et al., 2019a; Reeves et al., 2021). Although lacking the convenience of oral administration, rectal application is a simpler and more direct method of exposing the colorectal epithelia to the active pharmaceutical ingredient, with fewer concerns over drug stability, formulation, or exposure of nontarget tissues. Hence, the rectal route was selected for first-in-human administration to achieve clinical proof of principle, and our inclusion of a dose range-finding evaluation in the present study was designed to help select and justify a baseline clinical dose. Because EPICERTIN, like CTB, does not appear to be systemically absorbed, clinical safe starting doses can be better estimated from nonclinical topical exposures on the basis of anatomic comparisons among species (Permezel and Webling, 1971; Kararli, 1995; Casteleyn et al., 2010; Maynard and Downes, 2019). For the colon–rectum portion of gastrointestinal tract where UC pathology is mainly manifested, the surface area is 24 cm^2^ for mice (cylindrical area not taking into account microvillar surface area), 60 cm^2^ for rats, and about 3,000 cm^2^ for humans (Kararli, 1995.). Thus, the highest gastrointestinal exposure tested in mice of 61.4 µg (100 µL=l at 10 µM) EPICERTIN/administration would be equivalent to 2.6 µg drug/cm^2^ colorectal surface area (i.e., 61.4 µg/24 cm^2^). Similarly, for the rats administered EPICERTIN intrarectally in the dose-escalation study, the highest dose was 307 µg (1 ml at 5 µM)/60 cm^2^ colorectal surface, or 5.2 µg drug/cm^2^. The starting clinical doses currently contemplated are 368.5 µg (60-ml enema of a 0.1-µM EPICERTIN solution) and 1,100 µg (60 ml of a 0.3-µM drug solution), based on scaled volumes of administration and therapeutic responses seen in animal studies. A 60-ml enema volume is a clinical standard for UC drugs, such as mesalamine and Cortenema (https://dailymed.nlm.nih.gov/dailymed/). The high-dose patients would receive 1,100 µg drug/2,964 cm^2^ human colorectal surface, or 0.37 µg EPICERTIN/cm^2^. Based on these estimates and comparisons with animal dosages that showed no drug-related toxicity, the clinical margin of exposure at the highest clinical dose would be 7-fold (mouse) to 14-fold (rat) for all potentially exposed tissues, although further studies are necessary to evaluate drug distribution in the colorectal mucosa upon enema administration.

Recombinant CTB (rCTB) has been used for more than 20 years as an immunogen/adjuvant in the oral cholera vaccine Dukoral at 1 mg per dose, with two doses per regimen (prime/boost), or 2 mg total (Dukoral monograph, 2007; Sanofi Pasteur). rCTB has also been orally administered experimentally to Crohn’s disease patients in 5-mg doses, three times per week for 2 weeks, or 30 mg total (Stal et al., 2010). Similarly, rCTB has been orally administered experimentally to patients with Behçet's disease at 0.5-mg or 5-mg doses three times weekly for 12 weeks, or 180 mg total for the course of therapy (Stanford et al., 2004). Neither the vaccine nor the therapeutic studies cited reported adverse effects at any dose of CTB. We suspect that EPICERTIN, which differs from CTB in its KDEL-containing C-terminal addition, will be equally safe when assessed in UC and yet display wound-healing and tissue-repair activities not found in native CTB.

In conclusion, results of the current single-administration PK and acute safety studies in two species and verification of an optimal therapeutic dose in the DSS murine colitis model support further development of EPICERTIN as a novel biologic for ulcerative colitis.

## Abbreviations

AUC: area under the plasma concentration time curve
BA: bioavailability
*C*_max_: maximal observed plasma concentration
CRO: contract research organization
CTB: cholera toxin B subunit
DAI: disease activity index
DSS: dextran sodium sulfate
JVC: jugular vein catheterized
LLOQ: lower limit of quantitation
PK: pharmacokinetics
TK: toxicokinetic
T_max_: time to maximum plasma concentration
UC: ulcerative colitis
UPR: unfolded protein response
